# Topographic Patterns of Intracranial Meningioma Recurrences—Systematic Review with Clinical Implication

**DOI:** 10.3390/cancers16122267

**Published:** 2024-06-19

**Authors:** Sergio Corvino, Roberto Altieri, Giuseppe La Rocca, Amedeo Piazza, Giuseppe Corazzelli, Carmela Palmiero, Giuseppe Mariniello, Francesco Maiuri, Andrea Elefante, Oreste de Divitiis

**Affiliations:** 1 Department of Neurosciences, Reproductive and Odontostomatological Sciences, Neurosurgical Division, Università di Napoli Federico II, 80131 Naples, Italy; giuseppe.corazzelli@studenti.unina.it (G.C.); carmela.palmiero@unina.it (C.P.); giumarin@unina.it (G.M.); frmaiuri@unina.it (F.M.); oreste.dedivitiis@unina.it (O.d.D.); 2 Multidisciplinary Department of Medical-Surgical and Dental Specialties, University of Campania “Luigi Vanvitelli”, 80131 Naples, Italy; roberto.altieri.87@gmail.com; 3 Institute of Neurosurgery, A. Gemelli University Polyclinic, IRCCS and Foundation, Sacred Heart Catholic University, 20123 Rome, Italy; giuseppe.larocca@policlinicogemelli.it; 4 Department of Neurosurgery, “Sapienza” University, 00185 Rome, Italy; amedeo.piazza@uniroma1.it; 5 Department of Advanced Biomedical Sciences, School of Medicine, University of Naples “Federico II”, 80131 Naples, Italy; aelefant@unina.it

**Keywords:** intracranial meningiomas, recurrences, topographic pattern, classification, risk factors, radiation therapy

## Abstract

**Simple Summary:**

Meningiomas are the most common primary benign tumor of the central nervous system, and, despite their prevalent benign nature, they exhibit variable tendency to recur during the lifetime, even after multiple reoperations, adjuvant radiation treatment and several years, posing a significant challenge of management. While well-defined risk factors of recurrence have been identified, the topographic pattern after surgery has scarcely been investigated. Nevertheless, the possibility of theoretically predicting the site of recurrence provides a significant advantage for a multidisciplinary team during the decision-making process regarding the strategy of treatment at the first surgery. The authors performed a comprehensive and detailed systematic literature review on the topographic pattern of recurrence after surgical treatment for intracranial meningiomas.

**Abstract:**

Background: While several risk factors for recurrences have been defined, the topographic pattern of meningioma recurrences after surgical resection has been scarcely investigated. The possibility of theoretically predicting the site of recurrence not only allows us to better understand the pathogenetic bases of the disease and consequently to drive the development of new targeted therapies, but also guides the decision-making process for treatment strategies and tailored follow-ups to decrease/prevent recurrence. Methods: The authors performed a comprehensive and detailed systematic literature review of the EMBASE and MEDLINE electronic online databases regarding the topographic pattern of recurrence after surgical treatment for intracranial meningiomas. Demographics and histopathological, neuroradiological and treatment data, pertinent to the topography of recurrences, as well as time to recurrences, were extracted and analyzed. Results: Four studies, including 164 cases of recurrences according to the inclusion criteria, were identified. All studies consider the possibility of recurrence at the previous dural site; three out of four, which are the most recent, consider 1 cm outside the previous dural margin to be the main limit to distinguish recurrences closer to the previous site from those more distant. Recurrences mainly occur within or close to the surgical bed; higher values of proliferation index are associated with recurrences close to the original site rather than within it. Conclusions: Further studies, including genomic characterization of different patterns of recurrence, will better clarify the main features affecting the topography of recurrences. A comparison between topographic classifications of intracranial meningioma recurrences after surgery and after radiation treatment could provide further interesting information.

## 1. Introduction

Intracranial meningiomas account for more than one third of primary intracranial tumors [1,2] and pose a significant challenge for multidisciplinary management teams due to their propensity for recurrence [3]. In fact, despite their predominantly benign histology (80%), and although most patients recover following initial treatment [4], a not-insignificant percentage (about 20%) tends to have one or more recurrences over the patient’s lifetime [5,6,7,8], requiring additional treatments [4,9,10,11,12,13].

If, on the one hand, the advancements in the diagnostic and therapeutic tools over the years allow for better management of meningioma patients, resulting in a progressive improvement of the overall survival [14,15,16], on the other hand, the extension of life expectancy inexorably increases the risk of recurrence [5], which should be considered during the decision-making process of the strategy for treatment, as well as during the follow-up [5]. 

Management of meningioma recurrences often represents a challenge, especially when they occur in critical locations, close to highly functional neurovascular structures, like for skull base meningiomas. 

While over the years, many demographic, clinical, neuroradiological, pathological and surgical risk factors of recurrence have been identified [5,15,16,17,18,19,20,21,22,23,24,25,26,27,28,29,30,31,32,33,34,35,36,37,38,39], the topographic pattern has been scarcely investigated. Nevertheless, the possibility of theoretically predicting the site of recurrence is of paramount importance; in fact, the capability of knowing the spatial pattern of recurrences could not only help us to better understand the pathogenetic bases of recurrences and, thus, to drive the development of new targeted therapies, but also to assist the surgeon in the preoperative decision-making process in terms of surgical radicality and the radiation therapist in defining the surgical field to be included in the adjuvant treatment, in order to prevent/decrease the risk of recurrences. 

Therefore, the topography of meningioma recurrences represents a critical issue that deserves to be discussed. In the present study, we report on and compare, from a detailed and comprehensive literature review, the proposal classifications of intracranial meningioma recurrences according to their localization pattern after surgery. 

## 2. Materials and Methods

The MEDLINE and EMBASE online medical databases were screened in order to conduct a systematic review of the literature, according to the PRISMA statement [40], evaluating the topographic pattern of recurrence after surgical treatment for intracranial meningiomas. Records were searched for pertinent studies up to December 2023. We reviewed all abstracts of English-language articles containing the following keywords alone or in combination: “meningioma” AND “recurrent disease” AND “pattern”, “location”, “topography”, “recurrence pattern”, “multicentric”, “multifocal”, “multiple”. Each article of interest was marked for further review. The references listed in each paper were also reviewed for pertinent articles. The review of the titles and abstracts was conducted by two investigators (SC, IB). For studies that warranted full-text review, the same two reviewers evaluated each study independently. Any discordance in the screening process was solved by the consensus of two senior authors (GM, FM). From each study, demographic, histopathological, neuroradiological and treatment data, pertinent to the topography of recurrences, as well as time to recurrences, were extracted and analyzed. All extracted data were audited by the two independent auditors for accuracy and completeness.

The inclusion criteria were surgical series, reviews and case reports in English, reporting a classification of intracranial meningioma recurrences based on their topographic distribution detected on contrast-enhanced magnetic resonance imaging (MRI) were included. “Recurrence” was defined by intraoperative assessment of radical tumor removal (Simpson grades I and II) at first surgery and/or postoperative detection of no residual tumor on post-contrast brain MRI.

Articles describing the evaluation of other neoplasms besides intracranial meningiomas were not included. Reports of aggregated data and reports on multimodal therapy where surgery was not the primary treatment were excluded. In addition, exclusion criteria encompassed languages other than English, cadaveric studies and manuscripts not reporting a topographic classification. Duplicated papers were excluded from the screening. 

**Figure 1:**
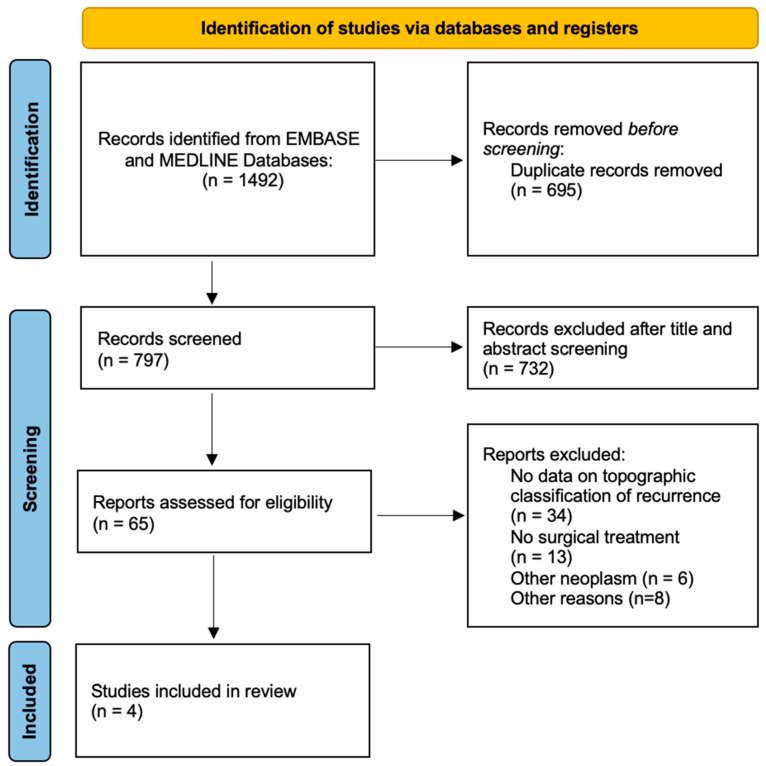
Flow chart showing the methods for the selection of the studies included in the review. Consider, if feasible to do so, reporting the number of records identified from each database or register searched (rather than the total number across all databases/registers). If automation tools were used, indicate how many records were excluded by a human and how many were excluded by automation tools. From [40]. For more information, visit: http://www.prisma-statement.org/.

In the second review round, papers included for full text analysis were screened and considered for inclusion according to the inclusion and exclusion criteria. The references of considered papers were then screened for papers erroneously missed in the first round of review (forward search). Papers not considered eligible were excluded with reason (Figure 1). Included papers were considered for data analysis and evidence synthesis. This study was conducted by following the Preferred Reporting Items for Systematic Reviews and Meta-Analyses (PRISMA) and in accordance with the Declaration of Helsinki. The study protocol was not registered. No new data were created or analyzed in this study. Data sharing is not applicable to this article.

## 3. Results

The literature review produced 1492 articles; after duplicate records were eliminated and papers were screened for title, abstract and full text reading, the remaining four studies [17,41,42,43] for a total of 164 cases of intracranial meningioma recurrences (Figure 1) were included in the review, and their reported features related to topographic pattern were analyzed and discussed.

### 3.1. Spatial Clustering Patterns of Intracranial Meningioma Recurrences According to Their Dural Origin or Attachment Based on Surgical Resection

**Figure 2:**
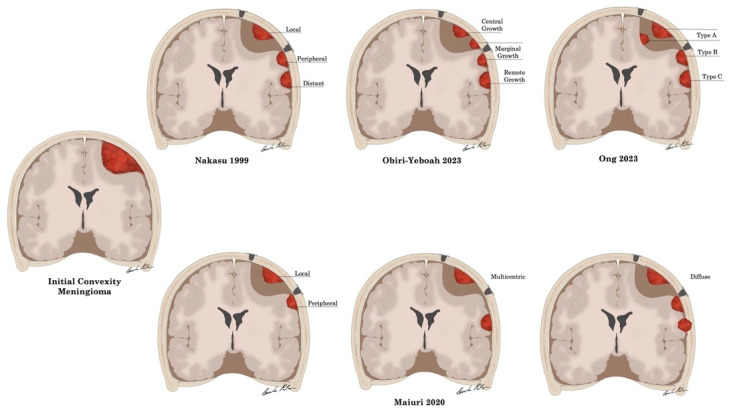
Artistic drawing [41,42,43]. Schematic representation of the topographic classifications of intracranial meningioma recurrences included in the systematic review.

To the best of our knowledge, four different topographic patterns of meningioma recurrences after surgical resection have been reported in the literature (Table 1 and Figure 2).

Nakasu et al. [41] classified meningioma recurrences into three types according their topographic pattern: “local recurrence”, at the same localization as the initial tumor; “peripheral recurrence”, in the dura adjacent to the site of tumor attachment, which was removed or coagulated; “distant recurrence”, ≥4 cm away from the original tumor. 

Maiuri et al. [17,44] classified intracranial meningioma recurrences, according to their appearance on contrast-enhanced magnetic resonance imaging and surgical description, into four types: Type 1, “local”, confined to the previous dural site; Type 2, “peripheral”, in the surrounding dura (within 1 cm), contiguous to the previous site; Type 3, “multicentric”, with multiple nodules both at the dural site and distant (>1 cm) from it, with seemly normal interposed dura mater; and Type 4, “diffuse”, with multiple nodules with interposed dural infiltration, or diffuse extradural infiltration; in addition, the authors further grouped these types into “local–peripheral” and “multicentric–diffuse”. 

Obiri-Yeboah et al. [42] defined recurrence as any radiographic progression and identified the following 3 zones of recurrence on T1-weighted volumetric contrast-enhanced magnetic resonance imaging: (1) “central growth”, observed inside the area of the previously resected tumor and more than 1 cm inside the original tumor margin; (2) “marginal growth”, observed within 1 cm (inside or outside) of the original tumor margin; and (3) “remote growth”, observed >1 cm outside the original tumor margin. 

Ong et al. [43] defined as recurrence the signs of tumor regrowth on follow-up imaging and classified recurrences in three types: Type A (within the surgical bed, which can be either on the dura side or on the brain side), type B (outside of the surgical bed but within 1 cm from the site), and type C (distal ≥ 1 cm of the resection site); in addition, the authors further defined recurrence types B and C as “beyond surgical bed” and type A as “within surgical bed”.

### 3.2. Analyzed Factors Affecting the Topographic Pattern of Recurrence after Surgery and Therapeutic Implication

Nakasu et al. [41] retrospectively reviewed the data of 101 patients with intracranial meningiomas with the aim of preoperatively identifying the clinical–radiological risk factors of recurrence; the authors disclosed 17 cases of recurrences: 9 were “local recurrences”, 7 were “peripheral recurrences”, and 2 were “distant recurrences”. One case exhibited a mixed pattern, local and peripheral. After investigating the extent of resection according to their Simpson score, WHO grade and proliferation index MIB1 in relationship with topography of recurrences, the authors observed that tumors with relatively higher proliferation potential recurred most often at the periphery of the dural resection and concluded asserting that meningiomas with mushrooming or lobulated shapes should be treated aggressively through a wider dural resection because recurrences more frequently occur at the edge of dural excision. 

Maiuri et al. [17,44], retrospectively analyzed data from 83 cases of intracranial WHO grade 1 and 2 meningioma recurrences, including 50 cases of “local–peripheral” recurrences and 33 cases of “multicentric–diffuse” recurrences, with the aim of defining the pathological and surgical risk factors correlated with both topographic patterns of recurrences. From the analysis of many demographic (sex and age), neuroradiological (tumor location and shape, brain–tumor interface), pathological (WHO grade, Ki67-MIB1, progesterone receptor expression), and surgical (entity of resection, number of reoperation) factors and time to recurrence and outcome, at initial diagnosis and at recurrence, revealed that the flat shape and values of Ki67 Li ≥ 4% were significantly associated with a higher risk of recurrence in multicentric–diffuse form. In addition, in terms of management and outcome, multicentric–diffuse recurrences were significantly associated with higher number of reoperations and lower rates of gross total tumor removal than local–peripheral forms. Finally, reoperations of multicentric–diffuse recurrences were significantly associated with better overall survival when compared to conservative treatment (no surgery) but showed lower rates of tumor control and higher rates of tumor progression and death than reoperations of local–peripheral recurrences. The authors concluded by asserting that even multiple reoperations over the years in selected patients with prevalent intradural tumor and not extensive “en-plaque” dural infiltration may obtain longer survival in non-anaplastic meningiomas.

Obiri-Yeboah et al. [42] performed a retrospective analysis of data of 22 patients who underwent surgical resection of a WHO grade 2 meningioma without adjuvant treatment and who experimented recurrence, including 19 cases of “central growth”, 21 cases of “marginal growth” and 8 cases of “remote growth”. Some tumors recurred in multiple zones. The aim of the authors was to characterize and classify the location of recurrences while also comparing them according to the extension of resection. After analyzing patient demographics, preoperative and recurrent tumor volume, location, pathology, extent of resection and time to recurrence, the authors found that most recurrences occurred centrally or at the margin of the initial tumor, while nearly one-third occurred in “remote” form regardless of the extent of resection. Therefore, the authors suggested including in dural resection or adjuvant radiation treatment at least 1 cm of dura beyond the dural margin of the initial tumor when an aggressive form of meningioma is suspected.

Ong et al. [43] reviewed data from 42 cases of intracranial WHO grade 1 meningiomas that were surgically resected and which experienced recurrence, including 28 cases (67%) of Type A, 11 cases (26%) of Type B and 3 cases (7%) of Type C, with the aim of investigating the spatial clustering pattern of recurrence relative to the original surgical bed. Analyzed factors at initial diagnosis included demographics, location, histologic grade and symptoms, whereas those at recurrence included location, modifications in histologic grade and clinical symptoms, time to recurrence and surgical features. No difference in spatial pattern of recurrence was registered in terms of Simpson grade or time to recurrence. The authors conclude by asserting that most intracranial grade 1 meningiomas have a tendency to recur within the surgical bed, whereas one-third recurred beyond the resection cavity; this knowledge might help to better understand disease progression and guide adjuvant therapy.

All these data are summarized in Table 2.

## 4. Discussion

After identifying solitary or multiple nodules protruding from the inner surface of the dura mater at a distance 1 to 3 cm from the site of attachment of the meningioma and microscopic islets of meningothelial cells within the internal layer or between the two layers of the dura mater, Borovich et al. [45] hypothesized the contribution of regional multicentricity to explain some unexpected recurrences. Therefore, based on these etiopathogenetic hypotheses, a wide resection of the dura mater, about 4 cm, surrounding the tumor attachment, configuring “Grade 0” of Simpson [30,46,47], was suggested to further decrease recurrence rate. Later, other authors observed tumor cell invasion over the tumor base and proposed the extension of dural resection for convexity [48] and falcine [49] meningiomas. Conversely, von Deimling et al. [50], in a small series of intracranial meningiomas, found that all the recurrences were clonal with respect to the primary lesions; thus, the authors concluded by asserting that most recurrent meningiomas were direct descendants of the primary lesions, arising locally from residual tumor or distantly from dissemination of tumor fragments either by surgical manipulation or by subarachnoid spread via cerebrospinal fluid. 

In recent years, many pre- and intraoperative tools to improve the extent of tumor resection while preserving neurological function [4,51,52,53,54,55,56,57,58] have been developed.

Meningiomas may recur not only within or close to the surgical bed, but also distant from it; therefore, the opportunity to theoretically predict at first diagnosis the possible site of recurrence is of paramount importance to guide treatment strategies, from both the surgical and the radiation therapy points of view, to decrease/prevent the recurrence risk and the complications related to repeated reoperations [10,11,12].

In this setting, several topographic patterns of intracranial meningioma recurrences after different radiation therapy techniques have been described [59,60,61,62,63] in terms of treatment failure, with the aim of better defining the risk factors of recurrence and to define the optimal target volume, boundaries of the field and radiation dose to increase the local and distant control of disease. Kuhn et al. [59] classified recurrences after stereotactic radiosurgery as “local failure” when tumor recurrence was either within the radiosurgical prescription volume (“central failure”) or just outside the radiosurgical prescription margin to within 2 cm of the tumor margin on MRI (“marginal failure”), and “distant failure”, when tumor recurrence occurred more than 2 cm outside the tumor margin on MRI; the authors found that multifocal disease and high-grade histology (WHO grades 2 and 3) were associated with higher risk of local recurrence; additionally, male sex was associated with higher risk of distant failure; finally, radiation doses greater than or equal to 12 Gy were associated with decreased local failure risk. Therefore, the authors suggested 12 Gy as the minimum sufficient margin dosage for management of meningiomas. Rajkrishna et al. [61] categorized recurrences after conformal radiation therapy administration as “in-field” when tumor recurrence occurred within the 90% isodose line, and “out-of-field” when tumor recurrence occurred outside the 90% isodose line; the authors concluded by suggesting that increased clinical target volume margin, escalated dose up to 59.4 Gy and 3D conformal RT may be helpful in preventing local recurrences in grade 2 and grade 3 meningiomas. 

While the topographic pattern of recurrence of intracranial meningiomas is largely addressed in the radiotherapy field, it is poorly investigated among the neurosurgical community, with only four studies [17,41,42,43] identified in the present systematic literature review and with heterogeneous analyzed factors. 

All included studies consider in the form of recurrence the site of the previous tumor. Three [17,42,43] out of the four studies, which also are the most recent, consider 1 cm outside the dural margin of the tumor the main limit to distinguish more peripheral or marginal recurrences from those more distant or remote.

Among the demographic risk factors for recurrence, none of the four studies identified a correlation between sex or age of patient and topography of recurrence.

Concerning neuroradiological findings related to recurrence risk, Nakasu et al. [41] found that skull base localizations only recurred locally or peripherally, but never distantly, while non-skull base meningiomas, in particular those of convexity, in a small percentage of cases also recurred distantly. This result can be justified by the more frequent distribution of low-grade meningiomas at the skull base versus not at the skull base [28]. Obiri et al. [42] and Maiuri et al. [17] did not find significant differences in terms of topography of recurrences according to initial tumor location. Nevertheless, Maiuri et al. [17] noted that flat-shaped morphology at initial diagnosis was associated with a significantly higher risk of multicentric–diffuse recurrence, while the loss of CSF–vascular cleft was not associated with topographic pattern of recurrence.

Proliferation index has been evaluated by Nakasu et al. [41] and Maiuri et al. [17], but its higher values were associated with “peripheral” recurrences in the classification provided by Nakasu et al. [41] and to the “multicentric–diffuse” recurrences in that one of Maiuri et al. [17] The mean value among those available for “peripheral recurrence” was 3.3%, while it was ≥4% for “multicentric–diffuse”. This result implies the need to adopt a common cut-off of Ki67-MIB1. Indeed, while the Ki67 proliferation index is widely accepted as a surrogate marker for recurrences and aggressive clinical behavior [64], and the value of 4% is frequently employed by most authors to dichotomize high and low risks for recurrence, the cutoff value still differs among groups of researchers. Maiuri et al. [17] also analyzed the correlations between WHO grade, as well as PR expression, and topographic pattern of recurrence, but no significant association was evidenced. 

In the study by Nakasu et al. [41], it is interesting to note that the only two cases of “distant” recurrences were Simpson grade I and WHO grade I, underlying that even complete tumor removal and apparently benign histological nature of the lesion, which represent the main good prognostic risk factors of recurrence, not only do not avoid the recurrences but are also associated with their occurrence distant from the site of initial tumor. Conversely, the gross total resection resulted significantly associated with higher risk of recurrence within the surgical bed in the studies of Obiri-Yeboah et al. [42] and Ong et al. [43]. In the study by Obiri-Yeboah et al. [42], the “remote” recurrences in one-third of cases both after gross total and subtotal resection was hypothesized to be due to tumor cells spreading through the vascular or lymphatic channels of meninges. 

Another interesting result and common finding resulting from all studies included in the review [17,41,42,43] is that there is no difference in time to recurrence [65] between the different patterns reported in each study.

Finally, the multicentric–diffuse pattern of regrowth at first recurrence, together with the extent of resection, is the main risk factor for multiple recurrences and reoperations [66].

The present literature review shows that most meningioma recurrences occur within or close to the surgical bed; nevertheless, up to one third may be detected beyond the resection cavity. Distant recurrences can occur regardless of Simpson score and WHO grade; flat-shaped morphology represents a factor associated with a more aggressive biological behavior. All these findings suggest a more aggressive surgical approach even for apparently benign lesions (based on preoperative neuroradiological findings and clinical course), by extending the resection of dura mater over the boundaries of the tumor attachment, up to 3 cm into apparently healthy surrounding dura. Indeed, despite recently some authors [67,68,69,70,71] have questioned the validity of the Simpson score as assessment method, the extent of resection is still one of the most important risk factors of recurrence. 

Further studies, focusing on the issue of topography of recurrences and analyzing more pathological, neuroradiological, surgical and molecular factors, are needed; moreover, in light of recent classifications of meningiomas and their recurrence risk based on molecular features [24,72,73,74], it would be useful to investigate the genomic characterization of different patterns of recurrence to guide postoperative strategy of treatment for intracranial meningiomas.

Last, but not least, a comparison between topographic classifications of intracranial meningioma recurrences after surgery and after radiation treatment could provide interesting information.

### Limits of the Study

Few studies are included in this systemic review, resulting in heterogeneous data in relation to the topography of recurrences. Statistical analysis was not possible due to the paucity and heterogeneity of available data.

## 5. Conclusions

Several demographic, histopathological, molecular, neuroradiological and treatment factors affect the recurrence risk of meningiomas. The ability to predict the site of recurrence at first diagnosis can not only result in a better understanding of the pathogenetic basis of disease and consequently drive development of new targeted therapy, but meanwhile can also assist the surgeon in the preoperative decision-making process in terms of surgical aggressivity as well as the radiation therapist in better targeting the surgical cavity and margins during adjuvant treatment in order to decrease the risk of recurrence and consequent reoperations and related complications. Unique topographic classification would be desirable.

## Figures and Tables

**Table 1:** Classification system of topographic pattern of intracranial meningioma recurrences after surgical resection.

Authors/Year	N. of Recurrences	Topographic Pattern of Recurrences
Nakasu et al. [41] 1999	17	*Local*: at the site of initial tumor *Peripheral*: adjacent to the site of initial tumor *Distant*: ≥4 cm away from the original tumor
Maiuri et al. [17] 2020	83	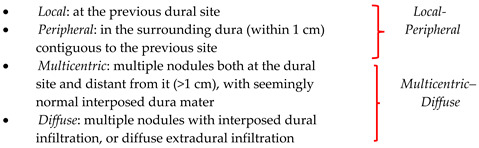
Obiri-Yeboah et al. [42] 2023	22	*Central growth:* at site of previous tumor, more than 1 cm inside the original tumor margin *Marginal growth:* within 1 cm (inside or outside) of the original tumor margin *Remote growth: >1 cm outside the original tumor margin.*
Ong et al. [43] 2023	42	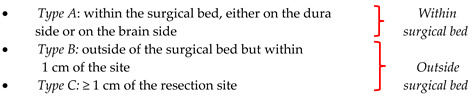

**Table 2:** Analyzed factors and treatment implication according to the topographic pattern of intracranial meningioma recurrences after surgery.

**Nakasu et al. 1999** [41]			
**Topographic Pattern of Recurrences**	**Significant Findings**		**Treatment Implication**
	Higher Proliferation Index—MIB 1		
**Local (9)**			Aggressive management for mushrooming or lobulated meningiomas
**Peripheral (7)**	+++		
**Distant (2)**			
**Maiuri et al. 2020** [17]			
**Topographic Pattern of Recurrences**	**Significant Findings**		**Treatment Implication**
	Flat-shape	Ki67 ≥ 4%	
**Local–peripheral (50)**			Multiple reoperations in selected patients may obtain longer survival in non-anaplastic meningiomas.
**Multicentric–diffuse (33)**	+++	+++	
**Obiri-Yeboah et al. 2023** [42]			
**Topographic Pattern of Recurrences**	**Significant Findings**		**Treatment Implication**
	GTR	STR	
**Central growth (19)**	+++	+++	Include in dural resection or adjuvant radiation treatment field at least 1 cm of dura beyond the dural margin of initial tumor when an aggressive form of meningioma is suspected.
**Marginal growth (21)**	+++	+++	
**Remote growth (8)**			
**Ong et al. 2023** [43]			
**Topographic Pattern of Recurrences**	**Significant Findings**		**Treatment Implication**
	Simpson Grade I-III		
**Type A (28)**	+++		Most recurrences occur in surgical bed and less frequently beyond surgical cavity; this knowledge might help us to better understand disease progression and guide adjuvant therapy.
**Type B (11)**			
**Type C (3)**			

Note that most of the studies employed in this review are very recent, as well as those addressing the topic of the management of multiple recurrences, underscoring how nowadays these topics are particularly important.

## Data Availability

Data from the current original research are available from the corresponding author on reasonable request.

## Abbreviations

WHO	World Health Organization
PR	Progesterone Receptor
MRI	Magnetic Resonance Imaging
RT	Radiation Therapy
